# Evaluation of the effects of a team‐based systematic prevention and management program for postoperative orthopedic older patients: A retrospective cohort study

**DOI:** 10.1002/pcn5.70021

**Published:** 2024-10-09

**Authors:** Fumitake Yamaguchi, Chie Inomata, Naoki Yoshinaga, Hirotake Sawada, Kazuko Shimamoto, Ayaka Haruta‐Tsukamoto

**Affiliations:** ^1^ School of Nursing, Faculty of Medicine University of Miyazaki Miyazaki Japan; ^2^ Department of Nursing University of Miyazaki Hospital Miyazaki Japan; ^3^ Department of Psychiatry, Division of Clinical Neuroscience, Faculty of Medicine University of Miyazaki Miyazaki Japan; ^4^ Nozaki Hospital Miyazaki Japan

**Keywords:** delirium, older patients, postorthopedic surgery, real‐world practice setting

## Abstract

**Aim:**

This study aimed to evaluate a team‐based systematic prevention and management program for delirium (a multicomponent intervention addressing potentially modifiable risk factors based on the DELirium Team Approach [DELTA]) in older patients undergoing orthopedic surgery within a real‐world clinical setting. The DELTA program was initiated at our hospital in January 2019.

**Methods:**

A retrospective before–after study was conducted during a preintervention period (January 1, 2017 to December 31, 2018) and a postintervention period (January 1, 2020 to December 31, 2021) at orthopedic wards of an advanced acute care hospital in Japan. A total of 787 inpatients were evaluated before the preintervention period, and 833 inpatients were evaluated after the postintervention period.

**Results:**

After the DELTA program's implementation, a significant decrease in benzodiazepine receptor agonist prescriptions (odds ratio [OR], 0.39; 95% confidence interval [CI], 0.29–0.53) and an increase in prescriptions of either melatonin receptor agonists or dual orexin receptor antagonists (OR, 3.83; 95% CI, 2.49–5.88) were observed. However, no significant difference was observed in the incidence of falls, self‐extubation, or required level of medical and nursing care, including risky behavior and inability to follow medical or care instructions following the intervention, despite a reduction in the length of hospital stay and institutionalization.

**Conclusion:**

Implementing the DELTA program for older patients undergoing orthopedic surgery contributed to optimizing the prescription of hypnotics; however, the impact on other patient outcomes, such as falls, self‐extubation, and required level of medical and nursing care was limited.

## Graphical abstract

This study aimed to evaluate a team‐based systematic prevention and management program for delirium, a multicomponent intervention addressing potentially modifiable risk factors based on the DELirium Team Approach (DELTA), in older patients undergoing orthopedic surgery within a real‐world practice setting. The results of this study suggest that implementing the DELTA in older patients undergoing orthopedic surgery contributed to optimizing the prescription of hypnotics; however, the impact on other patient outcomes, such as falls, self‐extubation, and required level of medical and nursing care, was limited.

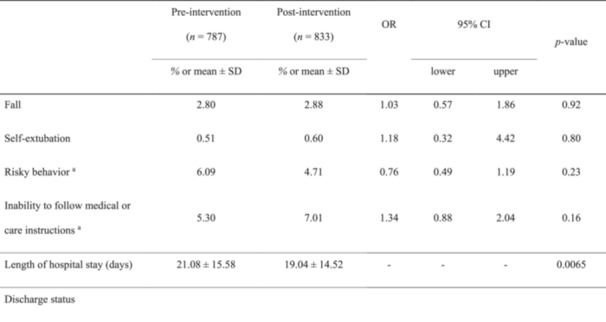

## INTRODUCTION

Delirium is an acute state of confusion characterized by disturbances of attention, awareness, and general cognition, and is characterized by rapid symptom changes, which pose challenges for healthcare providers who may find it difficult to anticipate its onset.^1^, ^2^ This can lead to incidents such as self‐extubation and removal of catheters,^3^ and falls.^4^, ^5^, ^6^ Delirium not only interferes with the treatment of the primary illness, but also leads to prolonged hospital stays,^7^, ^8^ increased mortality rate,^9^ and higher medical costs.^10^ Therefore, preventing and managing delirium is crucial for improving treatment outcomes and enhancing the quality of life (QOL) of patients and their families during and after hospitalization.^11^

Global aging trends, particularly acute in Japan's super‐aging society, have led to an increased incidence of delirium among hospitalized older patients, correlating with the increased frequency of surgeries due to medical advancements.^12^, ^13^ Among hospitalized patients over the age of 65, the incidence of delirium ranges from 10% to 30%,^14^, ^15^, ^16^ with even higher rates following surgery (12%––51%).^17^ This trend underscores the growing clinical and research interest in understanding, preventing, and managing delirium, particularly within an aging surgical population. This heightened prevalence poses challenges for patient management and highlights the broader implications of delirium on health systems and care strategies for older patients.

As there is little evidence supporting the efficacy of pharmacologic prevention of delirium, a great deal of attention has been devoted to nonpharmacological interventions that approach the complex factors associated with delirium.^18^, ^19^ Delirium can be caused by multiple factors, such as dehydration, infection, and electrolyte abnormalities, as well as by medications. Of the potentially modifiable risk factors, exposure to benzodiazepines (BZDs) appears to be the most strongly associated with delirium.^20^, ^21^ On the other hand, melatonin receptor agonists and dual orexin receptor antagonists (DORAs) have been reported to prevent the incidence of delirium.^22^, ^23^ A recent study reported several predictive factors (e.g., the type of surgery, multimorbidity, renal failure, polypharmacy, cut‐to‐suture time, and cognitive assessment).^24^ Embedding assessment in formal multifaceted structures, such as comprehensive geriatric assessment, may reduce postoperative delirium in older patients (e.g., those undergoing vascular or hip fracture surgery).^25^, ^26^

A growing body of evidence suggests that multi‐component interventions addressing potentially modifiable risk factors for delirium reduce the prevalence, incidence, and duration of the condition.^27^, ^28^ Implementing the DELirium Team Approach (DELTA), a multicomponent intervention for delirium prevention in patients hospitalized with cancer, was associated with a 48% reduction in the incidence of delirium (from 7.1% to 4.3%). Furthermore, the incidence of adverse events, including falls and self‐extubation, also decreased, from 3.5% to 2.6% (odds ratio [OR], 0.71; 95% confidence interval [CI], 0.54–0.92). Furthermore, there was a decrease in the prescription of BZDs (OR, 0.79; 95% CI, 0.71–0.87).^29^ Several studies have demonstrated the effectiveness of implementing team‐based, multicomponent interventions outside the oncology field for patients who had been admitted to a general‐medicine service at a teaching hospital, or to intensive care units.^27^, ^30^ However, reports on the effectiveness of a team‐based, systematic prevention and management program for delirium among older patients in the perioperative period of orthopedic surgery are limited. To the best of our knowledge, only one study reported a significant reduction in the incidence of delirium before and after the implementation of a multidisciplinary delirium prevention in postoperative orthopedic patients. However, that study did not examine whether adverse events (e.g., falls, tube removal) were reduced, or whether medication prescribing optimization (e.g., BZDs) was implemented.^31^

**Figure 1:**
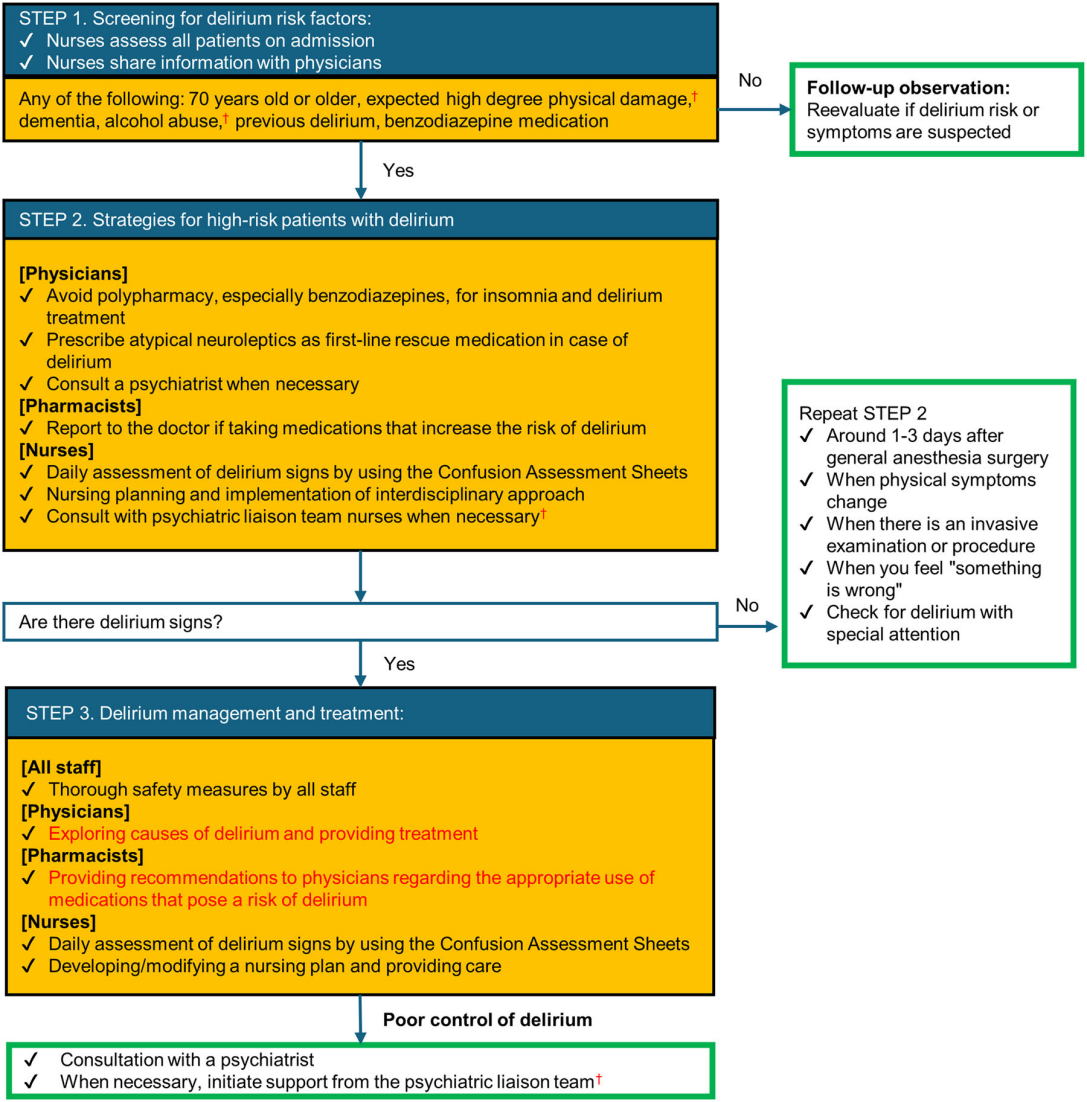
Flow of DELirium Team Approach (DELTA). The DELTA at our facility was adapted from the previously reported DELTA program created by the National Cancer Center. ^†^The previously reported DELTA program, created by the National Cancer Center,^29^ was modified to better suit our hospital's specific needs.

At our advanced acute care hospital, we initiated a DELTA program in January 2019, which was largely in line with previous studies but partially modified to fit our hospital setting.^29^ This program targets all hospitalized patients, aiming to screen high‐risk patients, prevent delirium through a standard care plan and interdisciplinary approach, which includes the appropriate prescribing of medications (e.g., avoid BZDs), and manage delirium by observing early symptoms and administering prescribed rescue medication (Figure 1).

Therefore, this study aims to evaluate the DELTA program, initiated in January 2019 at our hospital, for older patients undergoing orthopedic surgery in a real‐world practice setting. More specifically, we were keen to know whether the introduction of the DELTA program was associated with: (1) a decrease in the incidence of adverse events (e.g., falls and self‐extubation); (2) appropriate prescription of hypnotics (e.g., reduction of BZDs, and an increase of melatonin receptor agonists and/or DORAs); and (3) a decrease in risky behavior or inability to follow medical or care instructions within the first 3 postoperative days.

## METHODS

### Study design and setting

To evaluate the effectiveness of the DELTA program in older orthopedic perioperative patients, we conducted a retrospective cohort study before and after the intervention. The study phases were preintervention (January 1, 2017 to December 31, 2018) and postintervention (January 1, 2020 to December 31, 2021). The year 2019 was intentionally excluded from the study timeline to accommodate a pilot phase of the DELTA program and a dedicated period for dissemination among healthcare staff, ensuring a robust implementation from 2020. Patients admitted to the orthopedic ward of our advanced acute care hospital who had undergone surgery were included in the study. We excluded patients who were under 65 years of age, had not undergone surgery, and who opted out of participation. Data were extracted from medical charts and incident reports, each linked by patient ID.

### Implementing the DELTA program

Our DELTA program was initiated in 2019, prior to the start of additional medical fees for the care of patients at high risk for delirium under the Japanese national health insurance scheme revision in 2020. There have been no changes to the program following the start of the 2020 revision. The program was adapted from the previously reported DELTA program created by the National Cancer Center.^29^ However, the following minor modifications were made to the flow of DELTA to better suit the specific needs and situation of our hospital (Figure 1):

- As the only advanced medical facility with specialized functions in Miyazaki Prefecture, many patients require acute care. Therefore, we added the criterion “expected high degree of physical damage: requiring or scheduled for surgery under general anesthesia.”
- Given the high prevalence of heavy alcohol consumption in Miyazaki Prefecture,^32^ we implemented clear evaluation and judgment criteria for “alcohol abuse” based on the Alcohol Use Disorders Identification Test‐Consumption (AUDIT‐C) scoring system. Specifically, we defined alcohol abuse as an AUDIT‐C score of 5 or more for men and 4 or more for women.^33^
- Due to the involvement of multiple clinical departments, we added “Consult a psychiatrist or psychiatric liaison team nurses when necessary.”

Regarding staff education for the program, a one‐hour lecture‐based training for nursing staff was conducted in 2018; however, the attendance rate was relatively low (∼12%). For other healthcare professionals (physicians and pharmacists), written announcements/notices about the program were distributed via hospital conferences and the medical safety management manual.

### Outcomes

To assess the effectiveness of the DELTA program, we linked claims data and medical charts. The outcomes we collected/assessed were: (1) the incidence of adverse events, including falls and self‐extubation; (2) the use of BZDs (including both patients who were already taking BZDs prior to admission and those newly prescribed BZDs after admission) and melatonin receptor agonists or DORAs; and (3) the subscale of nursing care requirement, categorized as either “Risky behavior” or “Inability to follow medical or care instructions,” within the first 3 postoperative days; (4) the length of hospital stay; and (5) discharge status, including death.

Although some previous studies have reported chart‐based investigations into the incidence of delirium,^29^, ^34^ this study did not analyze the incidence of delirium as an outcome, due to the absence of a clear diagnosis of delirium prior to the implementation of DELTA in the medical record. Additionally, due to extraction limitations, we could not collect information about BZDs brought in by patients (i.e., those not prescribed by our hospital after admission).

The medical and nursing care requirement is an indicator used to objectively evaluate the condition of hospitalized patients in Japan. This indicator helps healthcare professionals assess and determine the necessary amount of medical and nursing care and facilitates the appropriate allocation of nursing staff. Several nurses assess patients according to a manual across more than 20 items during each shift. The evaluation items include the presence of risky behavior and the ability or inability to follow medical or care instructions.

### Statistical analysis

We compared the basic attributes of the patients before and after the intervention by χ^2^ analysis or Student's *t*‐test. To assess the balance of covariates, we used propensity scores. Clinical characteristics included sex, age, and fall risk score at admission. Using logistic regression models, we calculated and analyzed the OR for the outcomes after the implementation of DELTA. The significance level was set at 5%, with 95% confidence intervals. All statistical analyses were conducted using JMP Pro 14.2.0 (SAS Institute Inc.).

## RESULTS

### Demographic and baseline characteristics

Out of 4435 patients who underwent orthopedic surgery during the study period, 1620 patients met the selection criteria and were identified for analysis (787 preintervention, 833 postintervention). A comparison of patient categories before and after the intervention revealed a significant difference only in surgery duration (*p* = 0.0008; Table 1). However, propensity score analysis yielded a *c*‐statistic of 0.56, indicating that matching was not necessary.

**Table 1:** Demographic characteristics of pre‐ and postintervention groups.

	Preintervention (*n* = 787)		Postintervention (*n* = 833)		AR or diff	*p*‐value
	*n* (%) or mean ± SD		*n* (%) or mean ± SD			
Sex (female)^a^	417	(51.1)	399	(48.0)	0.0064	0.80
Age (years)^b^	74.63 ± 6.56		74.98 ± 6.78		0.35	0.29
Falls risk score on admission^b^	10.05 ± 3.98		10.05 ± 3.72		0.00001	0.99
Surgical duration (min)^b^	146.80 ± 119.77		130.56 ± 71.26		−16.29	0.0008

Abbreviations: AR, adjusted residuals for postintervention; diff, the difference in differences for postintervention; SD, standard deviation.

^a^ Fisher's exact test.

^b^ Student's *t*‐test.

### Incident reporting content

During the study period, 185 incidents were observed (70 preintervention, 115 postintervention). Of these, the total number of incidents was 119 (50 preintervention, 69 postintervention), with falls being the most common (23 [46.0%] preintervention and 25 [36.2%] postintervention). The occurrence of self‐extubation, including self‐removal of intravenous lines and indwelling urinary catheters, was four (8.0%) preintervention and five (7.2%) postintervention.

### Outcome evaluation

Logistic regression analysis (the presence of intervention as the independent variable, and the number of incidents of falls or self‐extubation as the dependent variable) demonstrated that the incidence rate of falls was 2.80% preintervention and 2.88% postintervention, with no statistical significance (OR, 1.03; 95% CI, 0.54–1.74). Similarly, there was no significant change in the incidence rate of self‐extubation, from 0.25% preintervention to 0.48% postintervention (OR, 1.89; 95% CI, 0.35–10.37). There were also no significant differences in risky behavior (OR, 0.76; 95% CI, 0.49–1.19) or inability to follow medical or care instructions (OR, 1.34; 95% CI, 0.88–2.04) based on the required level of medical and nursing care before and after the intervention. On the other hand, a significant decrease in BZD prescriptions (OR, 0.39; 95% CI 0.29–0.53) and an increase in prescriptions of either melatonin receptor agonists or DORAs (OR, 3.83; 95% CI 2.49–5.88) were observed after the intervention. Furthermore, after the introduction of the DELTA program, the length of hospital stay significantly decreased, and the proportion of patients discharged as outpatients significantly increased (OR, 1.36; 95% CI 1.11–1.67), while the rate of institutionalization (transfer to a nursing home or similar facility) significantly decreased (OR, 0.74; 95% CI 0.60–0.91). Additionally, there were two deaths among discharged patients before the intervention, while there were no deaths among discharged patients after the intervention (Table 2).

**Table 2:** Changes in outcomes after the implementation of the DELTA program.

	Preintervention (*n* = 787)	Postintervention (*n* = 833)	OR	95% CI		*p*‐value
	% or mean ± SD	% or mean ± SD		Lower	Upper	
Fall	2.80	2.88	1.03	0.57	1.86	0.92
Self‐extubation	0.51	0.60	1.18	0.32	4.42	0.80
Risky behavior^a^	6.09	4.71	0.76	0.49	1.19	0.23
Inability to follow medical or care instructions^a^	5.30	7.01	1.34	0.88	2.04	0.16
Length of hospital stay (days)	21.08 ± 15.58	19.04 ± 14.52	–	–	–	0.0065
Discharge status						
Discharged to home	30.75	37.70	1.36	1.11	1.67	0.0033
Discharged/transferred to another hospital or nursing home	69.00	62.30	0.74	0.60	0.91	0.0047
Death	0.25	0.00	‐	‐	‐	‐
Prescription of benzodiazepines	18.04	7.92	0.39	0.29	0.53	<0.0001
Prescription of a melatonin receptor agonist or dual orexin receptor antagonists^b^	16.53	43.11	3.83	2.49	5.88	<0.0001

A logistic regression model was used for binary data; Student's *t*‐test for “Length of hospital stay.”

Abbreviations: CI, confidence interval; OR, odds ratio.

^a^ Required level of medical and nursing care.

^b^ The denominator includes patients who were prescribed benzodiazepines, nonbenzodiazepine sleep medications, or new medications (melatonin receptor agonists or dual orexin receptor antagonists) during their hospital stay (total number of patients prescribed any of these medications: preintervention = 242; postintervention = 225).

## DISCUSSION

The introduction of the DELTA program resulted in a notable decrease in BZD prescriptions and an increase in prescriptions of either melatonin receptor agonists or DORAs. The reduction in BZD use observed in this study is important in Japan, where the consumption level of BZDs has been much higher than in other countries, and the problem of its long‐term use has long been discussed.^35^, ^36^, ^37^ A decrease in the prescription of BZDs may potentially lead to a reduction in the risk of their adverse effects, especially with respect to delirium.

Research indicates that alternative medications, such as melatonin receptor agonists and DORAs (instead of BZDs), may also improve delirium outcomes. A previous study demonstrated that the use of melatonin receptor agonists reduced the incidence of delirium in hospitalized patients.^38^ Similarly, findings by Matsuoka et al. suggest that DORAs can be effective in managing and preventing delirium.^23^ These findings suggest that the DELTA intervention, by reducing BZD prescriptions and potentially encouraging the use of safer alternatives, could improve delirium prevention and management.^39^ A decrease in BZD use and an increase in the use of melatonin receptor agonists or DORAs align with these alternative treatment approaches, which have been shown to be effective in the literature. Therefore, the DELTA program's impact on reducing BZD prescriptions and increasing the use of safer alternatives is likely a significant factor in improving delirium management in the studied patient population.

It is also important to note that the observed decrease in BZD prescriptions is not simply due to strict adherence to manual or protocol. In our hospital, “recommended medications” were provided as guidance, rather than mandating a specific change in prescriptions. The reduction in BZD use may reflect an increased awareness among physicians of the risks associated with delirium, leading them to proactively avoid prescribing BZDs. Pharmacists also played a crucial role by advising physicians to consider alternatives, such as orexin receptor antagonists. However, it is important to acknowledge that these changes in awareness and the influence of pharmacist advice were not quantified in this study. Therefore, these conclusions remain speculative.

Similar to previous studies,^17^, ^27^, ^29^, ^40^ the implementation of the DELTA program resulted in a significant increase in the proportion of outpatient discharges and a significant decrease in institutionalization (discharged/transferred to another hospital or nursing home) and length of hospital stay. These findings suggest that the DELTA intervention had a positive impact, because a longer duration of delirium among patients with critical illness is associated with mortality, increased length of hospital stay, greater healthcare costs, long‐term cognitive impairment, and disability in activities of daily living.^28^, ^41^ When comparing the average length of hospital stay for orthopedic beds during the study period with that of general beds at the study institution, in Miyazaki prefecture, and across Japan (Table 3),^42^, ^43^, ^44^ the average length of hospital stay at the University of Miyazaki Hospital has gradually decreased, particularly since 2019. Given that the DELTA program has been implemented across all wards at the study institution since 2019, it is reasonable to conclude that its introduction likely contributed to this reduction at the institutional level. Further research should investigate the impact of the DELTA program on other wards within the institution.

**Table 3:** Length of hospital stay during the study period for orthopedic beds in this study, compared to general beds at the study institution, in Miyazaki Prefecture, and across Japan.

	2017	2018	2019	2020	2021
University of Miyazaki Hospital—Orthopedic beds (our study)^a^	20.8	21.4		19.9	18.2
	(Preintervention)			(Postintervention)	
University of Miyazaki Hospital—General beds	14.6	14.5	14.0	13.9	12.8
Hospitals in Miyazaki prefecture—General beds	17.6	17.5	17.4	17.4	17.1
Hospitals in Japan—General beds	16.2	16.1	16.0	16.5	16.1

^a^ Data were based on the study sample of patients aged ≥65 years who had undergone orthopedic surgery.

Despite these positive changes (i.e., optimizing the prescription of hypnotics, shorter hospital stays, and deinstitutionalization), the primary outcomes indicating the occurrence of delirium, such as falls, self‐extubation, and the required level of medical and nursing care, were not significantly affected. In a previous report, the incidence of delirium in postoperative orthopedic patients was reported to be significantly reduced by a team‐based systematic prevention and management program.^31^ For this study, however, due to the lack of a clear diagnosis of delirium prior to the intervention, the incidence of delirium was not included as an outcome in the analysis. Instead, incidents mainly caused by delirium, such as falls and self‐extubation, and the required level of medical and nursing care were analyzed. These results suggest that while a multifaceted approach to delirium management may be effective, its direct preventive effects on postoperative delirium in orthopedic patients are limited. This further suggests that the direct preventive effects of the DELTA program on postoperative delirium in orthopedic patients are limited.

According to a meta‐analysis of non‐pharmacological interventions involving 1038 patients, the odds of falling were 62% lower in the intervention group compared with controls (OR, 0.38; 95% CI, 0.25–0.60).^45^, ^46^ Furthermore, a retrospective cohort study by Ogawa and colleagues on postoperative cancer patients reported a decrease in the incidence of falls from 2.1% to 1.3% following the implementation of the DELTA program (OR, 0.55; 95% CI, 0.32–0.93).^29^ One of the reasons for the different results obtained in this study compared to previous studies is the difference in patient characteristics. Ogawa et al. limited their study to cancer patients; including over 40% who were under the age of 65 years, and more than 90% who were independent in their activities of daily living (ADL) at the time of admission. In contrast, our study targeted older patients over 65 years who had undergone orthopedic surgery. Orthopedic diseases often interfere with mobility‐related ADL, and postoperative recovery of mobility‐related ADL is often a challenge. The patients of our study also included many patients who required assistance with ADL due to conditions such as osteoarthritis, femoral fractures, and spinal paralysis, with the average preoperative fall risk score exceeding Level II (requiring nurse intervention). Therefore, it is speculated that the overall rate of falls, which was already high in this group, was less likely to change. Regarding self‐extubation, since all patients included in the study required postoperative intravenous management and many required drain management, it is possible that this did not affect the frequency of tube‐related incidents. Several studies have reported various potential predictors related to postoperative delirium in orthopedic surgery, including age, gender, comorbidities, malnutrition, cognitive impairment, and specific drugs.^47^, ^48^ They suggest that further research is necessary to determine the most effective non‐pharmacological delirium‐prevention strategies for orthopedics. Patients undergoing surgery are more at risk for the onset of postoperative delirium due to enforced sleep by anesthesia during surgery, preoperative anxiety, postoperative pain, and insomnia due to mental state. In the future, it is necessary to verify how these factors are associated with the onset of delirium among older postoperative orthopedic patients.

This study has several limitations. The DELTA program used in this study was slightly modified to fit our hospital's specific setting, altering the content of screening, managing, and responding to delirium from the originally reported DELTA program.^29^ These modifications may have resulted in different effects compared to the original program.

Staff training was less intensive (only a few nurses received brief lecture‐based training) compared to the previous DELTA study that incorporated: (1) a 90‐min training session for nurses on prevention, screening, and treatment of delirium; (2) a 30‐min lecture for physicians and pharmacists on prevention and treatment of delirium; and (3) monthly case conferences for nurses.^29^ Insufficient staff education may have influenced the results.

The number and duration of physical restraints may impact the incidence of falls and self‐extubations, but due to the lack of standardized recording methods prior to the DELTA program, it was difficult to extract accurate data, making it challenging to investigate it thoroughly. Physical restraints are applied when necessary to protect the patient's life, yet recent studies suggest they do not effectively reduce the risk of falls.^49^ Therefore, examining the relationship between the number and duration of physical restraints and patient outcomes under the DELTA program would be meaningful.

Furthermore, due to the retrospective nature of the study, the incidence of delirium could not be clearly determined. The outcomes that could be investigated were limited, and there was a possibility of insufficient sample size due to the low number of major outcome incidents.

For future research, it is necessary to extend the study period and increase the number of outcomes to gather more comprehensive data, including length of stay and all procedural and pharmacy costs. Moreover, it is important to investigate whether the effects of the DELTA program vary based on factors such as the type of orthopedic disease, surgical procedures, and anesthesia used.

## CONCLUSION

Before and after implementing the systematic management program for delirium in postoperative orthopedic patients in a real‐world clinical setting, there was a decrease in BZD prescriptions and an increase in either melatonin receptor agonists or DORAs, which contributed to some improvements in delirium management. However, the impact on other patient outcomes, such as falls, self‐extubation, and the required level of medical and nursing care, was limited.

## AUTHOR CONTRIBUTIONS

Fumitake Yamaguchi, Chie Inomata, Naoki Yoshinaga, Hirotake Sawada, Kazuko Shimamoto, and Ayaka Haruta‐Tsukamoto contributed to the conception and design of this study. Fumitake Yamaguchi, Chie Inomata, Kazuko Shimamoto, and Ayaka Haruta‐Tsukamoto acquired data. Fumitake Yamaguchi, Chie Inomata, and Ayaka Haruta‐Tsukamoto performed the statistical analysis and drafted the manuscript. Kazuko Shimamoto, Hirotake Sawada, and Naoki Yoshinaga interpreted the data and critically reviewed the manuscript. Naoki Yoshinaga and Ayaka Haruta‐Tsukamoto supervised the whole study process. All authors read and approved the final manuscript.

## CONFLICT OF INTEREST STATEMENT

The authors declare no conflict of interest.

## ETHICS APPROVAL STATEMENT

This study was approved by the institutional research ethics board of the University of Miyazaki (approval number: 0‐1244). This study includes a retrospective chart review, making it difficult to obtain individual informed consent; hence, a waiver of informed consent was granted by the institutional research ethics board. However, an opportunity for opt‐out was provided by informing patients about the study and its purpose, through the study institution's website and by placing posters/leaflets at the relevant outpatient department.

## PATIENT CONSENT STATEMENT

This study includes a retrospective chart review, making it difficult to obtain individual informed consent; hence, a waiver of informed consent was granted by the institutional research ethics board. However, an opportunity for opt‐out was provided by informing patients about the study and its purpose, through the study institution's website and by placing posters/leaflets at the relevant outpatient department.

## CLINICAL TRIAL REGISTRATION

N/A.

## Data Availability

The data that support the findings of this study are available from the University of Miyazaki Hospital, although restrictions apply in regard to their availability. They were used under license for the current study and so are not publicly available. Data are available from the authors upon reasonable request and with permission of the University of Miyazaki Hospital.
